# High-resolution segmentation of the cavum septum pellucidum in young adult human brains

**DOI:** 10.3389/fnana.2025.1566762

**Published:** 2025-05-16

**Authors:** Andrew Rios, Achok Alier, Mihir Aneja, Farah Nimeri, Kayla Lavery, Jack Fisher, Rochana Wiyathunge, Marek Kubicki, Edward Yeterian, Sylvain Bouix, Nikos Makris, Hector Arciniega, R. Jarrett Rushmore

**Affiliations:** ^1^Department of Anatomy and Neurobiology, Chobanian and Avedisian School of Medicine, Boston University, Boston, MA, United States; ^2^Center for Morphometric Analysis, Department of Neurology and Psychiatry, Massachusetts General Hospital, Charlestown, MA, United States; ^3^Psychiatry Neuroimaging Laboratory, Brigham and Women’s Hospital, Boston, MA United States; ^4^Department of Psychology, Colby College, Waterville, ME, United States; ^5^Département de génie logiciel et TI | Software Engineering and IT, École de technologie supérieure, Université du Québec, Quebec City, QC, Canada; ^6^Department of Rehabilitation Medicine, New York University Grossman School of Medicine, New York, NY, United States; ^7^NYU Langone Concussion Center, NYU Langone Health, New York, NY, United States

**Keywords:** cavum septum pellucidum, high-resolution MRI, fifth ventricle, volumetric, sex differences

## Abstract

The cavum septum pellucidum (CSP) is a small cerebrospinal fluid-filled space found between the lateral ventricles of the forebrain that is often used as a biomarker for neurological disease and brain injury. The incidence of the CSP varies widely in different studies, with many reports finding that the CSP is frequently absent in healthy brains. Variables such as race, age and sex are typically not well-reported in CSP studies, presenting a challenge to understanding the normal distribution of the CSP in adult human brains. Moreover, the small size and frequently indistinct borders present a challenge for automated segmentation of the CSP. To address these issues, we developed a novel manual parcelation approach to volumetrically segment the CSP in high-resolution T1-weighted structural MRIs from male and female participants in the young adult dataset of the Human Connectome Project (HCP). We identified the CSP in 95.6% of subjects, compared to 57.1% when the automated segmentation approach was used on the same subjects. The CSP volume was significantly larger in male than female brains, both in terms of raw volume and volumes normalized for intracranial volume. To our knowledge, this study is the first to develop and validate a segmentation protocol for CSP volume, and to evaluate both the incidence and volume of the CSP in a representative population of young adults. Overall, these results provide a more accurate representation of the CSP in control populations, laying an improved foundation for its potential use as a biomarker for various disorders.

## Introduction

The cavum septum pellucidum (CSP), also known as the fifth ventricle, is a small space filled with cerebrospinal fluid located on the midline of the brain between the two lateral ventricles of the telencephalon (Schwidde, 1952; Rakic and Yakovlev, 1968; Sarwar, 1989; Tubbs et al., 2011). The CSP is separated from these ventricles laterally by the septum pellucidum and adopts a crescent shape posterior to the genu of the corpus callosum, which forms its superior, anterior, and inferior borders. The CSP is present during the fetal stages of development, but it is considered to disappear by 3 months of age (Rakic and Yakovlev, 1968).

The CSP does not appear to have a discrete function, but it has been investigated as a brain structure that may undergo volumetric changes in response to various disease states. It has been shown to increase in incidence and/or size in individuals with a variety of neurological and psychiatric disorders (see Supplementary Table 1), including chronic traumatic encephalopathy (CTE) (Alosco et al., 2021; Asken and Rabinovici, 2021), obsessive-compulsive disorders (Chon et al., 2010), psychopathy (White et al., 2013; Crooks et al., 2019), and schizophrenia (Degreef et al., 1992; DeLisi et al., 1993; Fukuzako et al., 1996; Shioiri et al., 1996; Kwon et al., 1998; Nopoulos et al., 1999; Filipović et al., 2004; Galarza et al., 2004; Flashman et al., 2007; Choi et al., 2008; Landin-Romero et al., 2016). The CSPs are also affected in clinical conditions such as infection (e.g., Akansel et al., 2003; Efendić et al., 2023) and cysts (Bikmaz et al., 2007). It has also been reported to increase in size and/or incidence in retired football players (Koerte et al., 2016; Arciniega et al., 2024), rugby players (Stanwell et al., 2022), and fighters (Bogdanoff and Natter, 1989; Lee et al., 2020). Thus, the CSP has been associated with brain injury or disease and has the potential to serve as a preclinical biomarker for various disorders.

To build on the potential of CSP as a biomarker, further research is needed to clarify its prevalence, size and persistence across diverse populations. This is important to better understand its role in brain pathology, and its potential as a diagnostic or prognostic marker. Current estimates of CSP incidence in control populations vary widely from 1.1% to 97.1% (see Supplementary Table 1), highlighting the need to determine its baseline prevalence for meaningful clinical comparisons. Moreover, many studies of CSP lack comprehensive demographic data such as sex, race or ethnicity, or rely on limited population samples, further complicating generalizability (see Supplementary Table 1). Additionally, differences in CSP assessment methods, such as semi-quantitative grading (e.g., absent, small, large), or midline length measurements limit meta-analytic integration despite internal validation through intra- and inter-rater reliability. The thin septum pellucidum, which borders the CSP laterally, presents challenges for volumetric segmentation, particularly when using low-resolution neuroimaging. Finally, automated segmentation techniques for CSP remain unreliable, and no large-scale studies using such automated approaches have yet measured CSP volume in the normal adult human brain.

The current study seeks to address these limitations by leveraging high-resolution structural magnetic resonance imaging (MRI) data from young adults as part of the Human Connectome Project [HCP; (Glasser et al., 2013)]. The high-resolution MRI scans enable more precise segmentation of the CSP than previously possible. Furthermore, the diverse and well-characterized demographic profile of the HCP dataset allows for a sample population to be selected, that is representative of the general United States population, addressing limitations of prior studies drawn from incomplete or unreported demographic representation (see Supplementary Table 1). To our knowledge, this study is the first to develop and validate a segmentation protocol for CSP volume, and to evaluate both the incidence and volume of the CSP in a representative population. In addition, we investigate sex differences, as previous research provided inconsistent findings (Schwidde, 1952; Jurjus et al., 1993; Born et al., 2004; de Souza Crippa et al., 2006; Chen et al., 2014). Overall, this comprehensive evaluation will provide a more accurate representation of the CSP in control populations, laying the groundwork for its potential use as a biomarker for various disorders.

## Methods

### Subjects

High-resolution structural MRI scans (*n* = 410) from the Human Connectome Project Young Adult Database (S1200) were used for this study (Glasser et al., 2013). ACPC-aligned T1-weighted scans (0.7 mm isotropic resolution) were visualized in 3D Slicer (version 5.2^1^), and the fifth ventricle was segmented using the neurosegmentation or segment editor modules (Fedorov et al., 2012; Rushmore et al., 2022). Subjects were selected from the HCP S1200 young adult database (206 females, 204 males), and the demographic variables of race and ethnicity were similar to the 2023 United States Census (Census Data Link), as shown in Table 1. Subjects were randomly selected based on demographic data and excluded if a cavum vergae was observed. Only one subject per family was chosen.

**TABLE 1:** Demographic distribution of the study population.

Demographic	Number of subjects	Age (SD)	Education [years (SD)]	BMI (SD)	Percentage in study	2023 U.S. census percentage
Asian and Pacific Islander	30 (15 Female)	26.6 (4.4)	16.0 (1)	22.6 (3.4)	7.3%	6.6%
Black or African American	49 (25 Female)	29.4 (3.5)	14 (2.1)	30.1 (6.6)	11.9%	11.8%
Hispanic	80 (39 Female)	27.6 (4.0)	14.8 (1.7)	27.0 (4.8)	19.5%	19.4%
Mixed race	17 (9 Female)	26.5 (3.8)	14.2 (1.5)	26.3 (5.6)	4.1%	4.4%
Other	2 (1 Female)	28.5 (9.2)	14 (2.8)	27.6 (2.3)	0.5%	0.6%
White	232 (117 Female)	29.0 (3.4)	15.2 (1.7)	26.3 (4.9)	56.6%	57.1%
Total	410 (206 Female)	28.5 (3.7)	14.9 (1.8)	26.6 (5.3)	100%	100%

### Segmentation of the cavum septum pelludicum

The CSP was manually segmented in each brain using a novel procedure developed to volumetrically segment the CSP (see Supplementary materials), which builds on the method developed by Rushmore et al. (2022) and validated through inter-rater and intra-rater Dice similarity coefficients. Volumes were calculated using 3D Slicer. In addition, automated calculations of the CSP, along with estimated intracranial volume, were compared in the same subjects using FreeSurfer (version 5.3), which was run as part of the HCP pipeline. It is important to note that FreeSurfer was modified extensively as part of the HCP pipeline (Glasser et al., 2013). Large CSPs that were continuous with the transverse cerebral fissure were classified as cavum vergae and those subjects with cavum vergae were excluded from the current study. Segmentation procedures were conducted by raters who were blinded to the subjects’ demographic information.

### Statistical analysis

The reliability of the segmentation procedure was evaluated by five different raters using Dice similarity coefficients. Given the small size of the fifth ventricle, a Dice coefficient larger than 0.8 was determined to be reliable. Statistical analysis was performed with JMP Pro17. Manual and automated segmentation methods were performed with a paired Student’s *t*-test. In the statistical tests, volume served as the dependent variable, with sex as the independent variable. Male and female CSPs were also normalized to head size using estimated total intracranial volumes (eTIV) obtained from the HCP FreeSurfer results. Since descriptive statistics of the raw and normalized volumetric data revealed skewed distributions (Shapiro-Wilk test: raw volumetric data - W = 0.633, *p* < 0.01; normalized volumetric data - W = 0.609, *p* < 0.01), Wilcoxon signed-rank tests were used to compare the raw and normalized CSP volumes between male and female brains. P values were considered significant if they were less than 0.05.

## Results

### Reliability

Manual segmentation of the CSP has proven challenging given its small size and variable incidence. In the current project, we developed a protocol for manual segmentation of the CSP (see Supplementary Data). Using this protocol, the average Dice similarity coefficient was 0.85 (SD = 0.066) with a median Dice coefficient of 0.85, indicating a strong inter-rater reliability.

### Incidence

**FIGURE 1:**
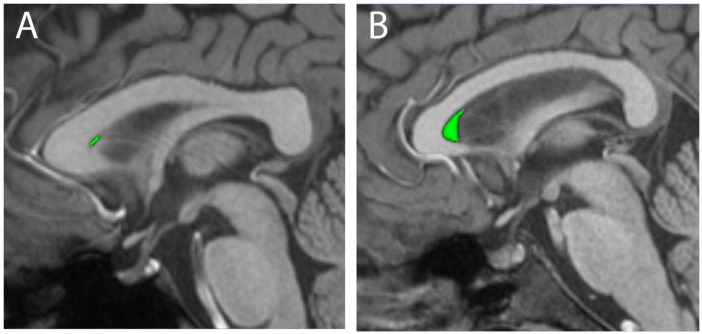
Three-dimensional renderings of manually segmented cava septa pellucida (CSP, green) are shown for representative small **(A)** and large **(B)** examples, overlaid on mid-sagittal T1-weighted MRI scans.

**FIGURE 2:**
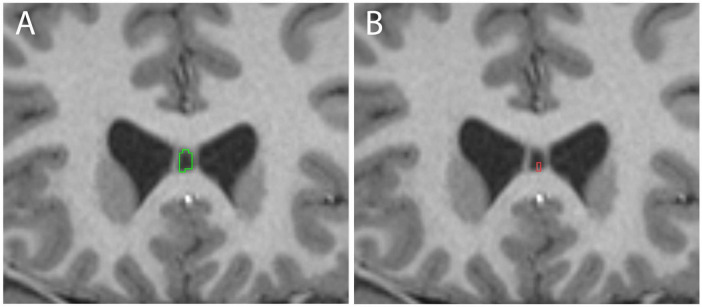
Examples of cavum septum pellucidum (CSP) delineation in the same subject using manual methods **(A**, green**)** and Human Connectome Project (HCP) FreeSurfer **(B**, red**)**.

The CSP was identified in nearly all cases (392/410 = 95.6%) using the manual segmentation protocol (Figure 1). The automated FreeSurfer program, used on the same 410 individuals, identified the CSP in 57.1% of cases (234/410). Visual inspection of automated segmentation results revealed inconsistencies, such that the CSP borders did not always align with the expected anatomical boundaries (Figure 2).

### Volume

The average volume of the CSP for the entire sample measured with the manual protocol was 17.2 mm^3^ [SD = 20.5; median = 11.5 mm^3^; 95% CI (15.2, 19.2); range: 0–214.7 mm^3^]. When excluding cases with zero values (i.e., including subjects with a present CSP), the mean volume increased to 18.0 mm^3^ [SD = 20.7 mm^3^; median = 12.3 mm^3^; 95% CI (15.9, 20.0); range: 0.7–214.7 mm^3^]. In contrast, the automated FreeSurfer segmentation protocol estimated the average CSP volume for the same subjects (including zero values) to be 4.1 mm^3^ [SD = 5.5; median = 1.8 mm^3^; 95% CI (3.6, 4.6), range 0–34.1 mm^3^]. After excluding zero values, the mean CSP volume from FreeSurfer was 7.2 mm^3^ [SD = 5.8; median = 5.6 mm^3^, 95% CI (6.5–7.9); range: 1.0– 34.1 mm^3^]. A paired *t*-test including all subjects (i.e., including 0 values) showed a significant difference between the results from the manual and automated methods [Manual mean = 17.2 mm^3^ FreeSurfer mean = 4.1 mm^3^; mean difference = 13.09, SD = 1.02, 95% CI (11.1, 15.1), t(409) = 12.78; *p* < 0.001)].

**FIGURE 3:**
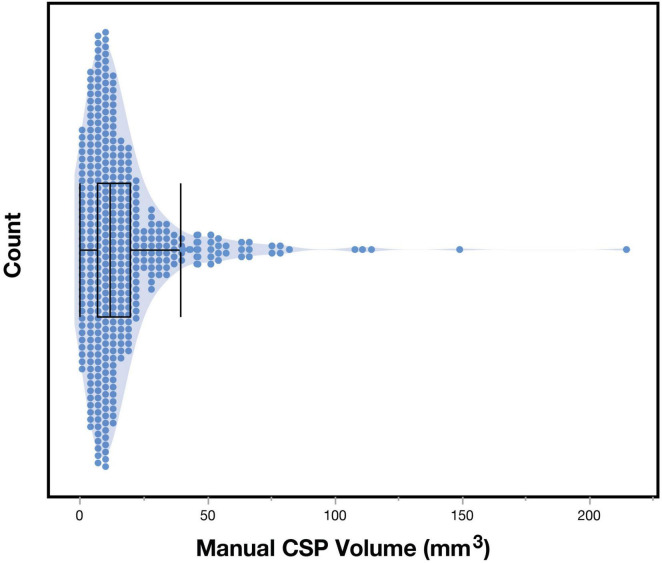
Distribution of cavum septum pellucidum (CSP) volumes. Figure show the histogram of manually-segmented CSP volumes (mm^3^) for the entire sample (*n* = 410).

The distribution of non-zero CSP volumes exhibited a rightward skew (Figure 3), with the peak distribution occurring between 5 and 10 mm^3^ (113/410 = 27%). While most of the sample had CSP values under 85 mm^3^, a small proportion (5/410 = 1.2%) exhibited notably large CSPs. We confirmed that the morphology of these large CSPs was not consistent with cavum vergae.

**FIGURE 4:**
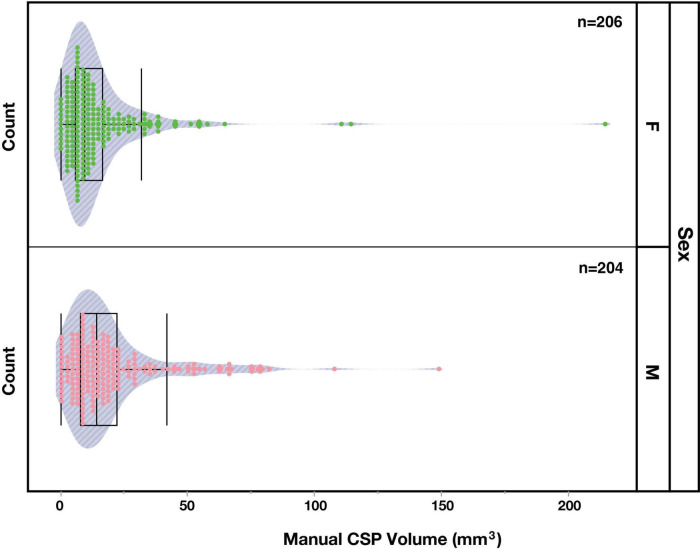
Distribution of manually segmented cavum septum pellucidum (CSP) volumes in the brains of female (F, green) and male (M, pink) subjects.

### Sex differences

The incidence of the CSP in male and female subjects was very similar. Of the 18 individuals who did not display an observable CSP, nine were female and nine were male. However, a Wilcoxon two-sample test revealed a sex difference (Z = -3.55, *p* < 0.001) between the CSP volumes of male (Mean = 19.5 mm^3^; SD = 20.2) and female (Mean = 14.9 mm^3^; SD = 20.6) subjects, indicating greater volumes in males. To account for potential confounding effects of brain size, volumes were normalized using eTIV. The normalized CSP volumes between males (Mean = 1.16e-5, SD = 1.19e-5) and females (Mean = 1.02e-5, SD = 1.44e-5) were significantly different (Wilcoxon Test: Z = 2.2, *p* = 0.027), indicating a greater volume of normalized CSP volumes in male subjects.

## Discussion

This study used a novel manual method to segment the CSP volumetrically in 410 young adult human high-resolution structural MRI scans. This sample was derived from the HCP young adult dataset, in proportions similar to the current demographic makeup according to the sex, race and ethnicity of the 2023 United States population. By using the high-resolution T1-weighted MRI data to visualize the CSP at a high level of detail, this study provides a thorough evaluation of the CSP incidence and volumetric variability within a diverse cohort of young adult brains. Additionally, our approach enabled the evaluation of sex differences. Below, we consider the key findings of our study and their substantial contributions to the existing body of research on the CSP.

### CSP manual segmentation protocol

We used a novel manual procedure to segment the CSP based on explicit anatomical definitions. Building on the method developed by Rushmore et al. (2022), this protocol was refined and validated. We found that our manual segmentation protocol identified the CSP in 95.6% of cases. In contrast, the automated FreeSurfer program identified the CSP in only 57.1% of cases. In quantifying volumetric measures, we also noted a difference between volumetric measures calculated by our manual and automated segmentation methods. The manual segmentations of the CSP yielded higher volumes than the FreeSurfer automated segmentations, a difference likely due to the fact that FreeSurfer was trained on lower-resolution MRI data and atlases. As a result, the CSP, and especially the smaller CSPs, may not have been visible in the training data. While these discrepancies in volumetric measurements highlight the challenges in achieving accurate automated segmentation of the CSP, it also suggests that more training data using higher resolution MRIs and anatomically precise CSP segmentations have the potential to improve the results of automated segmentation approaches.

### Sex differences

Previous studies examining sex differences in CSP have primarily focused on its incidence, reporting a higher prevalence in males compared to females. However, findings across studies have been inconsistent, reflecting variability in methodologies, sample populations, and definitions of CSP presence (Schwidde, 1952; Jurjus et al., 1993; Born et al., 2004; Crippa et al., 2004; Chen et al., 2014; Crooks et al., 2019). We evaluated sex differences in CSP volumes and found that males consistently had larger CSP volumes than females in both the raw and normalized values. This suggests that biological sex may contribute to CSP variability and should be considered as a relevant variable in future research. Notably, this difference persisted even after adjusting for brain size using eTIV, highlighting the robustness of the effect. These findings have implications for studies using the CSP as a potential biomarker.

### Comparison with previous studies

This study in a representative sample of young adult brains was motivated by the uncertainty regarding the incidence and volumetric characteristics of the CSP. To our knowledge, no studies have examined CSP volume in a large cohort of young adult individuals. As a result, comparisons of the present data to previous studies are challenging. However, prior research on CSP incidence has reported a broad range, from 1.1% to 97.1%. This wide variability may be attributed to the use of differing methodologies and subjects, such as the use of cadaveric specimens or low-resolution non-invasive imaging, as well as small sample sizes and unspecified comorbidities, all of which could contribute to the inconsistent incidence rates reported.

### Clinical considerations

A more thorough anatomical perspective has the potential to inform ongoing studies that examine CSP in disease. The CSP is widely studied in acute and chronic neurological and psychiatric disorders such as chronic traumatic encephalopathy (e.g., Alosco et al., 2021; Arciniega et al., 2024) and schizophrenia (e.g., Degreef et al., 1992; Landin-Romero et al., 2016). Findings are often reported as the degree to which the incidence of CSP in the clinical sample differs from that identified in a control sample. Our data suggest that CSP incidence is much higher than previously identified in control populations (Supplementary Table 1), a finding likely due to an inability to delineate smaller CSPs using low resolution scanning technologies. As high-resolution scanning becomes more accessible, more precise measurements of CSP volumes will be important in producing more refined measures of CSP in disease as a potential biomarker.

### Limitations and future directions

The use of high-resolution structural MRI scans enabled a more sensitive detection of the CSP, and allowed for a detailed assessment of its boundaries, leading to accurate volumetric estimates. The growing use of such high-quality imaging in both basic and clinical neuroscience is expected to improve the reliability and anatomical precision of CSP evaluations in these populations. Additionally, advancements in automated segmentation methods, trained on these high-resolution datasets, hold promise for further enhancing the accuracy and reliability of CSP segmentations. However, careful review and subsequent manual correction of the primary data should remain integral to any automated approach, particularly when delineating small structures like the CSP.

Based on our findings, comparison of CSP incidence rates between populations should be performed with caution. Studies examining the CSP typically compare incidence rates between a control and clinical population. Our approach revealed the presence of the CSP in 95.6% of our sample population, suggesting that a more sensitive analysis of the CSP may provide a limited dynamic range to detect and compare changes in the incidence rates. In addition, studies reporting changes in CSP incidence rates across different populations may have been limited by the resolution of MRI scans, and consequently the sensitivity of the imaging techniques used. While such comparisons are likely to be valid when conducted with consistent methods and within the sensitivity limits of the imaging resolution, attributing CSP expression to a specific factor may not be entirely accurate. With ongoing advancements in high-resolution structural MRI technology, future studies are likely to achieve greater anatomical clarity and precision, further refining our estimates of CSP incidence.

The rightward-skewed distribution of the present CSP volumes can be interpreted in two ways. First, it may reflect the distribution of the general young adult population. Alternatively, it could indicate that individuals with larger CSPs are in the early stages of disease or have experienced trauma, resulting in the expression of this space. However, the current study is unable to distinguish between these possibilities without additional data. Future research involving controlled populations and volumetric analysis may help to determine whether larger CSPs serve as indicators or predictors of clinical symptoms or traumatic events. Additionally, it is important to note that our findings are limited to young adults. Future research should aim to extend these results to middle-aged and older populations, enabling a deeper understanding of how age influences CSP characteristics and its potential clinical implications.

Another consideration in evaluating the CSP is the comparison of demographic characteristics between two or more groups. In many studies, particularly those with relatively low sample sizes, demographic factors may not always be evenly matched between groups. Our findings indicate that demographic factors such as sex, may play a significant role and should be carefully accounted for in future comparisons of CSP characteristics in normal and clinical populations.

## Conclusion

The results of this current analysis show that the CSP is more prevalent in the young adult human brain than previously reported. While most CSP volumes are small, there are notable instances of larger volumes as well. Additionally, we present normative data on CSP volumes in a well-characterized young adult population whose sex, racial and ethnic demographics closely mirror that of the United States population. We confirm that males exhibit a significantly larger CSP than females. Finally, we introduce a detailed protocol for manually segmenting the CSP and show that the use of automated approaches such as FreeSurfer may have challenges in capturing precise anatomical detail of the CSP.

## Data Availability

Publicly available datasets were analyzed in this study. The data can be found here; HP: https://www.humanconnectome.org/.

## Footnotes

1
www.slicer.org
