# Supplementary material for: High-resolution segmentation of the cavum septum pellucidum in young adult human brains
Source: Front Neuroanat. 2025 May 16;19:1566762. doi: 10.3389/fnana.2025.1566762 (PMC12131514; doi:10.3389/fnana.2025.1566762)
Supplement: Supplementary file 1 [file Data_Sheet_1.pdf]

<u>CSP Segmentation Protocol Using 3D Slicer</u>

Objective:

This protocol details the procedure for manually segmenting the **Cavum Septum Pellucidum (CSP)** in MRI scans using the program **3D Slicer**.

Step 1: Create New Segmentation

1.1 Open 3D Slicer and Add Data

- Launch **3D Slicer** on your workstation. At the top of the screen, click **File** and **Add data**.
- Choose the designated T1 image for upload (".....nii.gz file") and ensure **Volume** is selected under the description tab.

1.2 Select the Segment Editor Module

- From the **Module Finder**, search for and select the **Segment Editor** module, this is where all segmentation-related tasks will be performed.

1.3 Create a New Segmentation

- In the **Segment Editor**, click **Create new segmentation** to initialize the segmentation process.
- Select the current file name as the **Source volume.**
- Click **Add**, and rename your segment per the following naming convention:
  - "Segment_######_XX"
    - ###### is the file case number, XX will be your first and last initial

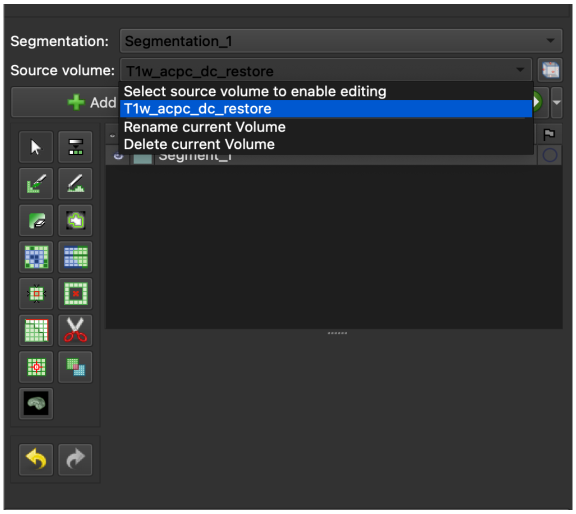

Figure 1

Step 2: Locate the CSP on coronal views

2.1 Find the Slice with the Largest CSP

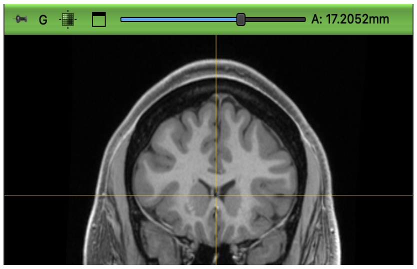

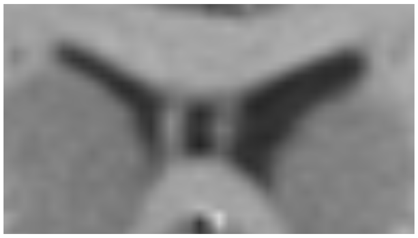

- Focus on the **coronal view** *(green)* and navigate through the slices.
- Locate the slice where the CSP appears largest and has the highest contrast compared to the surrounding regions.
- This slice will serve as your reference for initial thresholding.

Step 3: Apply Thresholding for Initial Segmentation

3.1 Open the Threshold Tool

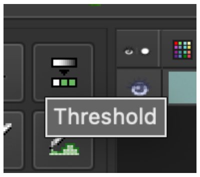

- In the **Segment Editor**, click the **Threshold** tool to define an intensity range for the CSP.

3.2 Applying Proper Threshold and Masking Region

- Navigate to the lower portion of the menu and expand the **Local Histogram** section.
- Select **Box** and use the cursor to select a portion of the CSP.
    - The portion selected should create a histogram with two distinct peaks on the low and high intensity ends of the histogram, and reflect an accurate voxel intensity composition per the visually confirmed CSP. The placement of the box should optimally include equal parts of the CSP and the adjacent structure. Finding the optimal position to place the histogram box will require trial and error. Note that separate histograms should be used for CSF-white borders and CSF-gray borders.

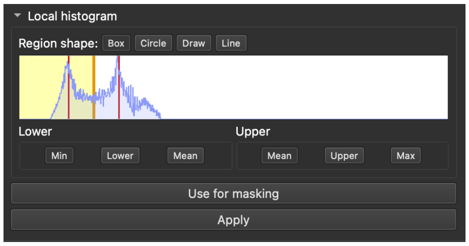

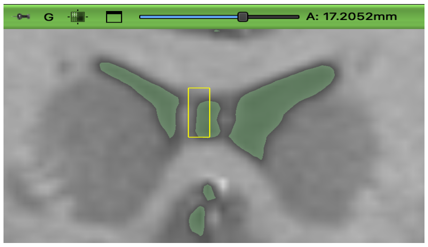

- Using the cursor, click on the vertical axis of the right peak within the histogram and drag until the cursor reaches the vertical axis of the left peak of the histogram.
- Select **Min** as the lower bound, and **Mean** as the upper bound.

Step 4: Create the Segment

4.1 Select Segmentation Tool

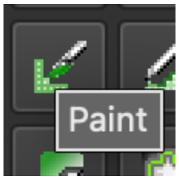

- Choose the **Paint Tool** in the Segment Editor and select the CSP voxels on the initial reference slide for thresholding.
- Navigate to slices both anterior and posterior of the initial reference slide. Complete the same process until the CSP has been fully segmented.

4.2 Review the Segmentation in Other Views

- The threshold approach will generate the initial segmentation, but viewing the CSP from different views may aid in finalizing the segmentation. It is important to turn off the interpolated view to identify the voxels directly.
    - **Enable/Disable Interpolated View** by clicking the pin icon followed by the double arrows, and lastly the interpolate background icon to enhance voxel visibility.
    - **Analyze the voxels from other viewpoints**: Look for unmarked regions that could be part of the CSP when seen on other views (sagittal, axial).

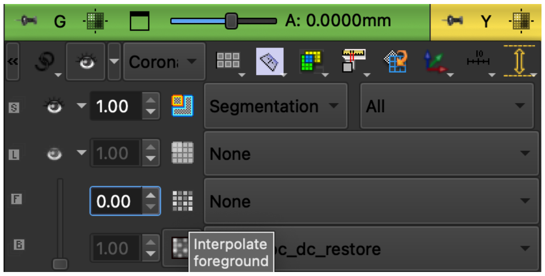

4.3 Check for Cavum Vergae and Questionable Voxels

- Large CSPs that do not appear to have a defined posterior border are considered as a separate category called the cavum vergae. Exclude such cases from segmentation of the CSP.

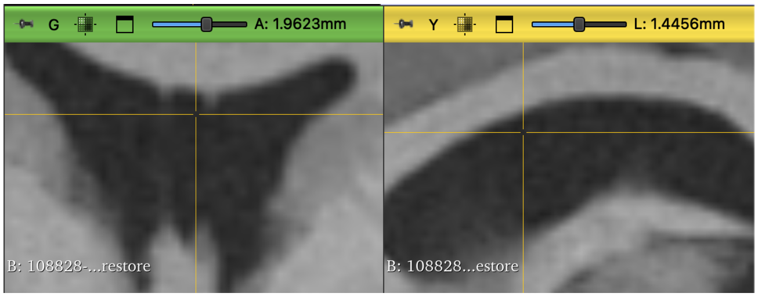

- If you identify voxels that are not continuous with the thresholded segment but are likely to be CSP, continue as follows.
    - Repeat the thresholding process detailed in steps 3.2 and attempt to isolate the questionable region using the threshold tool. If able to do so, select the voxel as part of the segment. If unable to confirm the aberrant voxel through thresholding, do not include it in the segment. However, take note for future discussion and peer review. To aid in this analysis, use the **crosshair tool** (top right of toolbar) to locate the voxel in all three views, and compare its intensity relative to surrounding tissue in different views.

Step 5: Refinement of the CSP

5.1 Examine Anterior-Posterior Regions

- Repeat the quality control process and focus on the **anterior** and **posterior** ends of the CSP. For both the anterior border and posterior borders, segmentation based on thresholding in the sagittal view may be useful; there are more voxels in this view that may allow for a better threshold estimate.

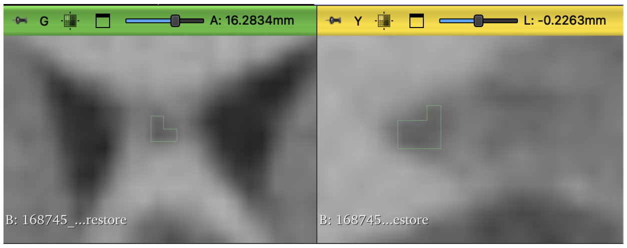

Step 6: Review and Finalize Segmentation

6.1 Inspect Segmentation and Validate Shape

- Review the segmented region in **coronal, sagittal, and axial views**, as well as the **3D model**. Zoom in and out.
- Inspect the overall shape of the segmented CSP in a non-interpolated view to ensure the segmentation creates contours without jagged or irregular boundaries in the CSP.
- Check for any inconsistencies, gaps, or regions where the segmentation does not match the expected anatomy (i.e., labeling voxels in the lateral ventricles).

- For questionable voxels attempt to confirm inclusion through above criteria. If any questions persist, take note for further discussion and agreement with study staff.

6.2 Use the Segment Statistics module to obtain a volume.

6.3 Save Segmentation

- Save the file using the following naming convention:
    - "######_CSP_XX"
        - ###### is the file case number, XX will be your first and last initials.
