# Supplementary material for: High-resolution segmentation of the cavum septum pellucidum in young adult human brains
Source: Front Neuroanat. 2025 May 16;19:1566762. doi: 10.3389/fnana.2025.1566762 (PMC12131514; doi:10.3389/fnana.2025.1566762)
Supplement: Supplementary file 2 [file Data_Sheet_2.pdf]

Author	Year	Method	Population	Overall Total (Count)	Control			Clinical				Male							Female						
													Control			Clinical				Control			Clinical		
					Incidence (Count)	Total (Count)	Incidence (Percentage)	Distribution	Incidence (Count)	Total (Count)	Incidence (Percentage)	Male Total	Incidence (Count)	Total (Count)	Incidence (Percentage)	Incidence (Count)	Total (Count)	Incidence (Percentage)	Female Total	Incidence (Count)	Total (Count)	Incidence (Percentage)	Incidence (Count)	Total (Count)	Incidence (Percentage)
Aldur et al.	1999	MRI	Control Only	505	7	505	1.4%					4		4					3		3				
Arciniega et al.	2024	MRI	Head Trauma (RHI); Football	405	26	55	47.3%		136	175	77.7%	240	26	55	47.3%	136	175	77.7%							
Aviv et al.	2010	MRI	Boxers	207	17	43	39.5%		81	164	49.4%														
Borgwardt et al.	2007	MRI	Psychosis	56	0	26	0.0%		1	30	3.3%														
Born et al	2004	MRI	Control Only	151	108	151	71.5%																		
Chen et al.	2014	CT	Control Only	19,031	5	19,031	0.03%																		
Choi et al.	2008	MRI	High Risk for Psychosis	87	33	34	97.1%		50	53	94.3%														
Chon et al.	2010	MRI	OCD	142	21	71	29.6%		43	71	60.6%	47				32	47	68.1%	24				11	24	45.8%
Crippa et al.	2004	MRI	Panic Disorder	42	18	21	85.7%		16	21	76.2%	10	10	10	100.0%				32	24	32	75.0%			
De Souza Crippa et al.	2006	MRI	Schizophrenia	76	3	38	8.9%		33	38	86.8%														
Degreef et al.	1992	Postmortem	Schizophrenia, Control	67	12	39	30.8%		17	28	60.7%														
Degreef et al.	1992	MRI	Schizophrenia, Control	127	1	46	2.2%		17	81	21.0%														
DeLisi et al.	1993	MRI	Schizophrenia	132	14	47	29.8%		38	85	44.7%														
Dickey et al.	2007	MRI	Schizotypal Personality Disorder	49	22	29	75.9%		17	20	85.0%														
Dunkel et al.	2017	MRI	Control HCP Data	526	287	526	54.6%																		
Filipovic et al.	2004	Postmortem	Head Trauma (RHI), Schizophrenia (SCHZ), Alcoholics (ALC)	110	4	40	10.6%	20 RHI, 25 SCHZ, 25 ALC		70	69.0% RHI, 83.3% SCHZ, 58.1% ALC														
Flashman et al	2007	MRI	Schizophrenia Spectrum	132	42	55	76.4%		53	77	68.8%														
Fukuzako et al.	1996	MRI	Schizophrenia	113	16	41	39.0%		34	72	47.2%														
Galarza et al.	2004	MRI	Schizophrenia	51	2	19	10.5%		14	32	43.8%														
Gardner et al.	2016	MRI	Football Players	34	11	17	64.7%		16	17	94.1%														
Hagino et al.	2001	MRI	Schizophrenia	165	59	79	74.7%		64	86	74.4%	90	32	46	69.6%	32	44	72.7%	75	32	40	80.0%	27	35	77.1%
Hughes et al.	1955	Postmortem		110	94	110	85.0%																		
Hwang et al.	2013	MRI	Opiate-dependent	132	43	67	64.2%		54	65	83.1%														
Jordan et al.	1992	CT		0																					
Jurjus et al.	1993	MRI	Schizophrenia, Control	104	7	37	18.9%		17	67	25.4%														
Karacan et al.	2021	MRI	Control Only	509	61	509	12.0%					34	19	34	55.7%				27	12	27	44.3%			
Kasai et al.	2004	MRI	Schizophrenia, Affective Psychosis	130	49	56	87.5%		56	74	75.7%														
Keshavan et al.	2002	MRI	Schizophrenia	99	7	59	11.9%		4	40	10.0%														
Kim et al.	2007	MRI	Bipolar	82	21	41	51.2%		28	41	68.3%														
Koerte et al.	2016	MRI	Football Players	86	8	14	57.1%		66	72	91.7%														
Kwon et al	1998	MRI	Schizophrenia, Affective Disorder, Schizotypical	113	39	46	84.8%		49	67	73.1%														
Landin-Romero et al.	2016	MRI	Psychotic or Mood Disorders	862	71	223	31.8%		314	639	49.1%	99	37	99	37.4%				124	34	124	27.4%			
Lee et al.	2020	MRI	Fighters	500	11	63	17.5%		217	437	49.7%														
Myslobodsky et al.	1995	MRI	PTSD	31	3	21	14.3%		5	10	50.0%														
Nopoulos et al.	1997	MRI	Schizophrenia	130	44	75	58.7%		32	55	58.2%	76	51	76	67.1%				54	35	54	64.8%			
Oktem et al.	2018	MRI	Control Only	3128	117	3128	3.7%																		
Rajarethinam et al	2001	MRI	Schizophrenia	116	18	43	41.9%		44	73	60.3%				33.0%			57.0%				50.0%			70.0%
Rajarethinam et al.	2008	MRI	Schizophrenia, High Risk for Psychosis	273	77	120	64.2%		98	153	64.1%														
Schidde et al.	1952	Postmortem		1032	206	1032	20.0%																		
Scott et al.	1993	MRI/CT	Schizophrenia, Control	131	0	79	0.0%		0	52	0.0%														
Shaw & Alvord	1969	Postmortem		374	45	374	12.0%																		
Shiori et al.	1996	MRI	Bipolar, MDD, Schizophrenia	132	1	92	1.3%		7	40	17.5%														
Shunk	1963	Postmortem		307	184	307	60.0%																		
Takahashi et al.	2010	MRI	Bipolar	364	133	163	81.6%		157	201	78.1%														
Takahashi et al.	2008	MRI	FEP, Schizophrenia, High Risk	338	78	87	89.7%		223	251	88.8%														
Takahashi et al.	2009	MRI	Major Depressive Disorder	89	28	33	84.8%		48	56	85.7%														
Takahashi et al.	2007	MRI	Schizophrenia, Schizotypal	50	18	24	75.0%		22	26	84.6%														
Trzesniak et al.	2012	MRI	Schizophrenia (SCHZ), Mood Disorders (MD),Other Psychosis (OP)	216	83	94	88.3%	62 SCHZ, 46 MD, 14 OP	115	122	94.3%														
Tsutsumi et al.	2017	MRI	Control Only	60	25	60	41.7%																		
Van Wagenen & Aird	1934	Postmortem	Control Only	30	18	30	60.0%																		

Supplementary Table 1 References

Aldur, M. M., Gürcan, F., Başar, R., & Akşit, M. D. (1999). Frequency of septum pellucidum anomalies in non-psychotic population: a magnetic resonance imaging study. *Surgical and Radiologic Anatomy: SRA*, *21*(2), 119–123.

Arciniega, H., Jung, L. B., Tuz-Zahra, F., Tripodis, Y., John, O., Kim, N., Carrington, H. W., Knyazhanskaya, E. E., Chamaria, A., Breedlove, K., Wiegand, T. L. T., Daneshvar, D., Billah, T., Pasternak, O., Coleman, M. J., Adler, C. H., Bernick, C., Balcer, L. J., Alosco, M. L., … DIAGNOSE CTE Research Project. (2024). Cavum septum pellucidum in former American football players: Findings from the DIAGNOSE CTE Research Project. *Neurology. Clinical Practice*, *14*(5), e200324.

Aviv, R. I., Tomlinson, G., Kendall, B., Thakkar, C., & Valentine, A. (2010). Cavum septi pellucidi in boxers. *Journal l'Association Canadienne Des Radiologistes [Canadian Association of Radiologists Journal]*, *61*(1), 29–32; quiz 1–2.

Borgwardt, S. J., Radue, E.-W., & Riecher-Rössler, A. (2007). Cavum septum pellucidum in patients with first episode psychosis and individuals at high risk of psychosis. *European Psychiatry: The Journal of the Association of European Psychiatrists*, *22*(4), 264.

Born, C. M., Meisenzahl, E. M., Frodl, T., Pfluger, T., Reiser, M., Möller, H. J., & Leinsinger, G. L. (2004). The septum pellucidum and its variants. An MRI study. *European Archives of Psychiatry and Clinical Neuroscience*, *254*(5), 295–302.

Chen, J.-J., Chen, C.-J., Chang, H.-F., Chen, D.-L., Hsu, Y.-C., & Chang, T.-P. (2014). Prevalence of cavum septum pellucidum and/or cavum Vergae in brain computed tomographies of Taiwanese. *Acta Neurologica Taiwanica*, *23*(2), 49–54.

Chon, M.-W., Choi, J.-S., Kang, D.-H., Jung, M. H., & Kwon, J. S. (2010). MRI study of the cavum septum pellucidum in obsessive-compulsive disorder. *European Archives of*

Psychiatry and Clinical Neuroscience, *260*(4), 337–343.

Crippa, J. A. S., Uchida, R., Busatto, G. F., Guimarães, F. S., Del-Ben, C. M., Zuardi, A. W., Santos, A. C., Araújo, D., McGuire, P. K., & Graeff, F. G. (2004). The size and prevalence of the cavum septum pellucidum are normal in subjects with panic disorder. *Brazilian Journal of Medical and Biological Research*, *37*(3), 371–374.

Degreef, G., Bogerts, B., Falkai, P., Greve, B., Lantos, G., Ashtari, M., & Lieberman, J. (1992a). Increased prevalence of the cavum septum pellucidum in magnetic resonance scans and post-mortem brains of schizophrenic patients. *Psychiatry Research*, *45*(1), 1–13.

Degreef, G., Bogerts, B., Falkai, P., Greve, B., Lantos, G., Ashtari, M., & Lieberman, J. (1992b). Increased prevalence of the cavum septum pellucidum in MRI scans and postmortem brains of schizophrenic patients. *Schizophrenia Research*, *6*(2), 145.

DeLisi, L. E., Hoff, A. L., Kushner, M., & Degreef, G. (1993). Increased prevalence of cavum septum pellucidum in schizophrenia. *Psychiatry Research. Neuroimaging*, *50*(3), 193–199.

de Souza Crippa, J. A., Zuardi, A. W., Busatto, G. F., Sanches, R. F., Santos, A. C., Araújo, D., Amaro, E., Hallak, J. E. C., Ng, V., & McGuire, P. K. (2006). Cavum septum pellucidum and adhesio interthalamica in schizophrenia: an MRI study. *European Psychiatry: The Journal of the Association of European Psychiatrists*, *21*(5), 291–299.

Dickey, C. C., McCarley, R. W., Xu, M. L., Seidman, L. J., Voglmaier, M. M., Niznikiewicz, M. A., Connor, E., & Shenton, M. E. (2007). MRI abnormalities of the hippocampus and cavum septi pellucidi in females with schizotypal personality disorder. *Schizophrenia Research*, *89*(1-3), 49–58.

Dunkel, C. S., Cho, H. B., van der Linden, D., Michael Woodley of Menie, & Platek, S. M. (2017). Cavum septum pellucidum volume and life history strategy. *Evolutionary Behavioral Sciences*, *11*(3), 262–271.

Filipović, B., Prostran, M., Ilanković, N., & Filipović, B. (2004). Predictive potential of cavum septi pellucidi (CSP) in schizophrenics, alcoholics and persons with past head trauma. A

post-mortem study. *European Archives of Psychiatry and Clinical Neuroscience*, *254*(4), 228–230.

Flashman, L. A., Roth, R. M., Pixley, H. S., Cleavinger, H. B., McAllister, T. W., Vidaver, R., & Saykin, A. J. (2007). Cavum septum pellucidum in schizophrenia: clinical and neuropsychological correlates. *Psychiatry Research*, *154*(2), 147–155.

Fukuzako, T., Fukuzako, H., Kodama, S., Hashiguchi, T., & Takigawa, M. (1996). Cavum septum pellucidum in schizophrenia: a magnetic resonance imaging study. *Psychiatry and Clinical Neurosciences*, *50*(3), 125–128.

Galarza, M., Merlo, A. B., Ingratta, A., Albanese, E. F., & Albanese, A. M. (2004). Cavum septum pellucidum and its increased prevalence in schizophrenia: a neuroembryological classification. *The Journal of Neuropsychiatry and Clinical Neurosciences*, *16*(1), 41–46.

Hagino, H., Suzuki, M., Kurokawa, K., Mori, K., Nohara, S., Takahashi, T., Yamashita, I., Yotsutsuji, T., Kurachi, M., & Seto, H. (2001). Magnetic resonance imaging study of the cavum septi pellucidi in patients with schizophrenia. *The American Journal of Psychiatry*, *158*(10), 1717–1719.

Hughes, R. A., Kernohan, J. W., & Woltman, H. W. (1955). Abscess of cavum septum pellucidum: report of a case. *Proceedings of the Staff Meetings. Mayo Clinic*, *30*(23), 536–538.

Hwang, J., Kim, J. E., Kaufman, M. J., Renshaw, P. F., Yoon, S., Yurgelun-Todd, D. A., Choi, Y., Jun, C., & Lyoo, I. K. (2013). Enlarged cavum septum pellucidum as a neurodevelopmental marker in adolescent-onset opiate dependence. *PloS One*, *8*(10), e78590.

Jordan, B. D., Jahre, C., Hauser, W. A., Zimmerman, R. D., Zarrelli, M., Lipsitz, E. C., Johnson, V., Warren, R. F., Tsairis, P., & Folk, F. S. (1992). CT of 338 active professional boxers. *Radiology*, *185*(2), 509–512.

Jurjus, G. J., Nasrallah, H. A., Olson, S. C., & Schwarzkopf, S. B. (1993). Cavum septum

pellucidum in schizophrenia, affective disorder and healthy controls: a magnetic resonance imaging study. *Psychological Medicine*, *23*(2), 319–322.

Karacan, K., & Karacan, A. (2021). Investigation of septum pellucidum and its variations with magnetic resonance imaging. *Journal of Surgery and Medicine*. https://doi.org/10.28982/josam.798360

Kasai, K., McCarley, R. W., Salisbury, D. F., Onitsuka, T., Demeo, S., Yurgelun-Todd, D., Kikinis, R., Jolesz, F. A., & Shenton, M. E. (2004). Cavum septi pellucidi in first-episode schizophrenia and first-episode affective psychosis: an MRI study. *Schizophrenia Research*, *71*(1), 65–76.

Keshavan, M. S., Jayakumar, P. N., Diwadkar, V. A., & Singh, A. (2002). Cavum septi pellucidi in first-episode patients and young relatives at risk for schizophrenia. *CNS Spectrums*, *7*(2), 155–158.

Kim, M. J., Lyoo, I. K., Dager, S. R., Friedman, S. D., Chey, J., Hwang, J., Lee, Y.-J., Dunner, D. L., & Renshaw, P. F. (2007). The occurrence of cavum septi pellucidi enlargement is increased in bipolar disorder patients. *Bipolar Disorders*, *9*(3), 274–280.

Koerte, I. K., Hufschmidt, J., Muehlmann, M., Tripodis, Y., Stamm, J. M., Pasternak, O., Giwerc, M. Y., Coleman, M. J., Baugh, C. M., Fritts, N. G., Heinen, F., Lin, A., Stern, R. A., & Shenton, M. E. (2016). Cavum septi pellucidi in symptomatic former professional football players. *Journal of Neurotrauma*, *33*(4), 346–353.

Landin-Romero, R., Amann, B. L., Sarró, S., Guerrero-Pedraza, A., Vicens, V., Rodriguez-Cano, E., Vieta, E., Salvador, R., Pomarol-Clotet, E., & Radua, J. (2016). Midline brain abnormalities across psychotic and mood disorders. *Schizophrenia Bulletin*, *42*(1), 229–238.

Lee, J. K., Wu, J., Bullen, J., Banks, S., Bernick, C., Modic, M. T., Ruggieri, P., Bennett, L., & Jones, S. E. (2020). Association of cavum septum pellucidum and cavum vergae with cognition, mood, and brain volumes in professional fighters. *JAMA Neurology*, *77*(1), 35–

42.

Myslobodsky, M. S., Glicksohn, J., Singer, J., Stern, M., Bar-Ziv, J., Friedland, N., & Bleich, A. (1995). Changes of brain anatomy in patients with posttraumatic stress disorder: a pilot magnetic resonance imaging study. *Psychiatry Research*, *58*(3), 259–264.

Nopoulos, P., Swayze, V., Flaum, M., Ehrhardt, J. C., Yuh, W. T., & Andreasen, N. C. (1997). Cavum septi pellucidi in normals and patients with schizophrenia as detected by magnetic resonance imaging. *Biological Psychiatry*, *41*(11), 1102–1108.

Oktem, H., Dilli, A., Kurkcuoglu, A., & Pelin, C. (2018). Prevalence of septum pellucidum variations: A retrospective study. *OAlib*, *05*(11), 1–9.

Rajarethinam, R., Miedler, J., DeQuardo, J., Smet, C. I., Brunberg, J., Kirbat, R., & Tandon, R. (2001). Prevalence of cavum septum pellucidum in schizophrenia studied with MRI. *Schizophrenia Research*, *48*(2-3), 201–205.

Rajarethinam, R., Sohi, J., Arfken, C., & Keshavan, M. S. (2008). No difference in the prevalence of cavum septum pellucidum (CSP) between first-episode schizophrenia patients, offspring of schizophrenia patients and healthy controls. *Schizophrenia Research*, *103*(1-3), 22–25.

Schunk, H. (1963). Congenital dilatations of the septum pellucidum. *Radiology*, *81*(4), 610–618.

Schwidde, J. T. (1952). Incidence of cavum septi pellucidi and cavum Vergae in 1,032 human brains. *A.M.A. Archives of Neurology and Psychiatry*, *67*(5), 625–632.

Scott, T. F., Price, T. R., George, M. S., Brillman, J., & Rothfus, W. (1993). Midline cerebral malformations and schizophrenia. *The Journal of Neuropsychiatry and Clinical Neurosciences*, *5*(3), 287–293.

Shaw, C.-M., & Alvord, E. C., Jr. (1969). CAVA SEPTI PELLUCIDI ET VERG Æ: THEIR NORMAL AND PATHOLOGICAL STATES. *Brain: A Journal of Neurology*, *92*(1), 213–224.

Shioiri, T., Oshitani, Y., Kato, T., Murashita, J., Hamakawa, H., Inubushi, T., Nagata, T., & Takahashi, S. (1996). Prevalence of cavum septum pellucidum detected by MRI in patients

with bipolar disorder, major depression and schizophrenia. *Psychological Medicine*, *26*(2), 431–434.

Stanwell, P., Iverson, G. L., Van Patten, R., Castellani, R. J., McCrory, P., & Gardner, A. J. (2022). Examining for cavum septum pellucidum and ventricular enlargement in retired elite-level rugby league players. *Frontiers in Neurology*, *13*, 817709.

Takahashi, T., Suzuki, M., Hagino, H., Niu, L., Zhou, S.-Y., Nakamura, K., Tanino, R., Kawasaki, Y., Seto, H., & Kurachi, M. (2007). Prevalence of large cavum septi pellucidi and its relation to the medial temporal lobe structures in schizophrenia spectrum. *Progress in Neuro-Psychopharmacology and Biological Psychiatry*, *31*(6), 1235–1241.

Takahashi, T., Yücel, M., Lorenzetti, V., Nakamura, K., Whittle, S., Walterfang, M., Suzuki, M., Pantelis, C., & Allen, N. B. (2009). Midline brain structures in patients with current and remitted major depression. *Progress in Neuro-Psychopharmacology and Biological Psychiatry*, *33*(6), 1058–1063.

Takahashi, T., Yung, A. R., Yücel, M., Wood, S. J., Phillips, L. J., Harding, I. H., Soulsby, B., McGorry, P. D., Suzuki, M., Velakoulis, D., & Pantelis, C. (2008). Prevalence of large cavum septi pellucidi in ultra high-risk individuals and patients with psychotic disorders. *Schizophrenia Research*, *105*(1-3), 236–244.

Trzesniak, C., Schaufelberger, M. S., Duran, F. L. S., Santos, L. C., Rosa, P. G. P., McGuire, P. K., Murray, R. M., Scazufca, M., Menezes, P. R., Hallak, J. E. C., Crippa, J. A. S., & Busatto, G. F. (2012). Longitudinal follow-up of cavum septum pellucidum and adhesio interthalamica alterations in first-episode psychosis: a population-based MRI study. *Psychological Medicine*, *42*(12), 2523–2534.

Tsutsumi, S., Ishii, H., Ono, H., & Yasumoto, Y. (2018). Visualization of the cavum septi pellucidi, cavum Vergae, and cavum veli interpositi using magnetic resonance imaging. *Surgical and Radiologic Anatomy: SRA*, *40*(2), 159–164.

Van Wagenen, W. P., & Aird, R. B. (1934). Dilatations of the cavity of the septum pellucidum

and cavum vergae: Report of cases. *American Journal of Cancer*, *20*(3), 539–557.
